# Attenuated SARS-CoV-2-Specific T Cell Responses Are Associated with T Follicular Helper Cell Expansion in Treatment-Naive Chronic Lymphocytic Leukemia Patients

**DOI:** 10.3390/pathogens14090890

**Published:** 2025-09-05

**Authors:** Baiba Šlisere, Roberts Kārkliņš, Alla Rivkina, Sandra Lejniece, Kristīne Oļeiņika

**Affiliations:** 1 Department of Internal Diseases, Rīga Stradiņš University, LV-1007 Riga, Latvia; 2 Chemotherapy and Hematology Clinic, Rīga East University Hospital, LV-1038 Riga, Latvia; 3 Joint Laboratory, Pauls Stradiņš Clinical University Hospital, LV-1002 Riga, Latvia

**Keywords:** chronic lymphocytic leukemia, SARS-CoV-2 immune response, T follicular helper cells, T follicular regulatory cells

## Abstract

Chronic lymphocytic leukemia (CLL) is associated with immune dysfunction, but how disease-intrinsic mechanisms in treatment-naive patients influence the coordination of adaptive responses to novel antigens remains unclear. Here, we assessed SARS-CoV-2-specific antibody and T cell immunity in 38 treatment-naive CLL patients and 13 healthy controls (HCs) following vaccination. Despite significantly reduced total immunoglobulin levels compared to HCs, 94.7% of CLL patients developed SARS-CoV-2-specific IgG, and 89.5% mounted IgA responses, with serum titers comparable to those of HCs. Virus-specific T cell responses, measured by IFN-γ release following antigen stimulation, were detected in 78.9% of patients. CLL patients had significantly more circulating CD4^+^ T follicular helper (Tfh) and T follicular regulatory (Tfr) cells than HCs. These expansions correlated with B cell abundance, which, in untreated CLL, predominantly reflects malignant B cells. Notably, Tfh cell frequencies and absolute counts were highest in patients lacking a SARS-CoV-2-specific T cell response, indicating a decoupling between Tfh expansion and functional antiviral immunity. Overall, these findings demonstrate that while SARS-CoV-2-specific immune responses are largely preserved in treatment-naive CLL patients, disease-driven alterations in T cell composition may compromise the coordination and quality of antigen-specific T cell-mediated immunity.

## 1. Introduction

Chronic lymphocytic leukemia (CLL) is a malignancy of mature B cells that predominantly affects older adults [1,2]. The neoplastic B cells characteristically co-express CD5, CD19, CD23, and have low expression of CD20 and surface immunoglobulin [1,3]. These clonally expanded B cells accumulate in secondary lymphoid organs, blood, and bone marrow [4].

CLL is characterized by immune dysfunction that arises because of both tumor-driven mechanisms and treatment-related effects [2,5,6,7]. This dysregulation predisposes CLL patients to infectious complications, which remain a leading cause of morbidity and mortality [2,5,8]. This vulnerability was starkly highlighted during the COVID-19 pandemic [9,10], when CLL patients experienced higher SARS-CoV-2-related mortality compared to the general population. Although vaccination remains the cornerstone of SARS-CoV-2 prevention, impaired immune competence is thought to contribute to the variable—and often suboptimal—vaccine responses in this population [7,11,12,13,14,15]. Emerging evidence suggests that some patients—particularly those who are treatment-naive, in remission after therapy, or receiving venetoclax monotherapy—can mount SARS-CoV-specific antibody and T cell responses [16]. However, it remains challenging to disentangle the effects of the disease itself from treatment-related immune suppression, as untreated individuals are typically underrepresented in published cohorts.

Effective antiviral immunity relies on coordinated yet distinct contributions from both B and T cells. High-affinity antibody production depends on germinal center (GC) formation, where B cells undergo somatic hypermutation and affinity maturation [17]. These processes are critically supported by T follicular helper (Tfh) cells—a specialized CD4^+^ T cell subset that expresses CXCR5 and localizes to B cell follicles. Tfh cells promote B cell activation, class switching, and plasma cell differentiation [17,18]. T follicular regulatory (Tfr) cells, a subset of FoxP3^+^ regulatory cells, share phenotypic features with Tfh cells, and co-localize in the GC, where they suppress excessive Tfh activity and constrain B cell responses [19]. In parallel, virus-specific effector T cells—including cytotoxic CD8^+^ and Th1-polarised CD4^+^ subsets—play a critical role in directly eliminating infected cells and orchestrating antiviral immune responses.

In CLL, there are widespread disruptions in immune architecture. Hypogammaglobulinemia [2,7,20,21], loss of naive B cells [22], and changes in T cell composition and function, including in Tfh cells [23,24,25,26,27,28], are common even in early disease. However, the functional implications of these changes on antigen-specific immune responses remain poorly defined, particularly in untreated patients.

Because Tfh–B cell collaboration and effector T cells are both essential for antiviral defense, we investigated how immune dysregulation in treatment-naive CLL patients affects their capacity to mount SARS-CoV-2-specific antibody and T cell responses. Specifically, we examined how global immune alterations—including reduced total serum immunoglobulin levels and circulating Tfh and Tfr cell expansions—relate to virus-specific immunity.

Despite widespread antibody and T cell abnormalities, many treatment-naive CLL patients mounted SARS-CoV-2-specific immune responses. Notably, Tfh cells were most expanded in patients who failed to generate a virus-specific T cell response, and their frequencies correlated with B cell abundance, reflecting the predominantly malignant B cell compartment in CLL. Our findings reveal a broader dissociation between immune architecture and antigen-specific immunity, with signs of functional disruption in a subset of patients.

## 2. Materials and Methods

### 2.1. Study Participants and Sample Collection

This study was approved by the Medical and Biomedical Research Ethics Committee of Rīga East University Hospital (approval no. 7-A/21) and conducted in accordance with the Declaration of Helsinki. Written informed consent was obtained from all participants. Between January 2022 and August 2023, 38 CLL patients were prospectively enrolled at the Chemotherapy and Hematology Clinic of Rīga East University Hospital, where peripheral blood samples were collected during routine visits, and 13 age- and sex-matched healthy controls (HCs) were recruited in parallel. Inclusion criteria for all participants were age ≥18 years and SARS-CoV-2 vaccination prior to sampling. Inclusion criteria for CLL patients were a confirmed diagnosis of CLL according to the WHO 2017 classification and International Workshop on CLL (iwCLL) criteria [29,30], with all patients treatment-naive (newly diagnosed or under “watch-and-wait” management). At the time of sampling, most patients had early-stage disease (Binet stage A) according to the Binet classification [31]. Exclusion criteria for CLL patients included any prior CLL-directed therapy, such as anti-CD20 monoclonal antibodies or Bruton’s tyrosine kinase inhibitors (BTKi). Additional exclusions were immunoglobulin replacement therapy within the preceding 6 months (based on published estimates of immunoglobulin (Ig) half-life), autoimmune conditions not associated with CLL, and concurrent secondary neoplasia. Exclusion criteria applied to both patients and controls were acute infection and primary immunodeficiency. HCs were required to be without hematological malignancy or ongoing immunosuppressive therapy. Information on adverse events following vaccination and the clinical course of prior SARS-CoV-2 infection was not systematically collected. However, available medical records were reviewed, and no evidence of vaccine-related adverse events, severe COVID-19, or related complications was documented for any of the study participants. The demographic, clinical, and laboratory characteristics of the study participants are summarized in Table 1.

### 2.2. Quantification of Total and SARS-CoV-2-Specific Serum Immunoglobulins

Total serum IgM, IgG, and IgA were measured by turbidimetry using Siemens systems (Erlanger, Germany). SARS-CoV-2-specific IgG and IgA antibodies were detected in serum samples by ELISA (EI 2606-9601-10 G and EI 2606-9601 A, Euroimmun, Luebeck, Germany). These kits used the recombinant S1 domain of the SARS-CoV-2 spike protein (Wuhan-Hu-1 isolate) as the antigen. Results were evaluated semi-quantitatively by calculating a ratio of the extinction of the participant sample over the extinction of the calibrator. Interpretation was carried out in accordance with Euroimmun recommendations (negative < 0.8; borderline 0.8–1.1; positive ≥ 1.1). Previous natural exposure to SARS-CoV-2 was assessed by measuring serum antibodies against SARS-CoV-2 nucleocapsid protein (NCP) using an ELISA kit (EI 2606-9601-2 G, Euroimmun).

### 2.3. Assessment of Functional T Cell Responses to SARS-CoV-2

SARS-CoV-2-specific T cell responses were assessed using an IFN-γ release assay (IGRA) with the QuantiFERON SARS-CoV-2 blood collection tubes and QuantiFERON SARS-CoV-2 ELISA kit (Qiagen, Hilden, Germany), following the manufacturer’s instructions. 1 mL of lithium-heparinized whole blood was added to each of the four tubes provided in the kit: Nil (negative control), Ag1, Ag2 (SARS-CoV-2 peptide antigens), and Mitogen (positive control). Tubes were incubated at 37 °C for 20 ± 4 h, after which they were centrifuged, and plasma was harvested and stored at −20 °C until used for ELISA analysis. To correct for background responses, the IFN-γ concentration in the Nil tube was subtracted from that in the Ag1, Ag2, and Mitogen tubes. The Ag1 tube contains peptides that predominantly stimulate CD4^+^ T cells, while the Ag2 tube contains peptides recognized by both CD4^+^ and CD8^+^ T cells. The assay cutoff was IFN-γ level ≥ 0.15 IU/mL. A positive T cell response was defined as an IFN-γ level ≥ 0.15 IU/mL in the Ag2 tube, after background subtraction. Ag2 was chosen because this peptide pool includes epitopes that stimulate both CD4^+^ and CD8^+^ T cells. Thus, it provides the broadest and most sensitive composite readout of SARS-CoV-2-specific cellular immunity.

### 2.4. Peripheral Blood Mononuclear Cell Isolation

Peripheral blood mononuclear cells (PBMCs) were isolated from lithium-heparinized whole blood by density gradient centrifugation using SepMate tubes (Stemcell, Hannover, Germany), and subsequently cryopreserved at −80 °C until flow cytometry analysis.

### 2.5. Flow Cytometry

PBMCs were thawed and up to 2.5 × 10^6^ cells per sample were stained with LIVE/DEAD™ Fixable Near-IR Dead Cell Stain Kit (Invitrogen, Carlsbad, CA, USA) for 10 min at room temperature. After washing, FcR blocking reagent (Miltenyi Biotec, Bergisch Gladbach, Germany) was added for 10 min at 4 °C to reduce nonspecific binding. Cells were then incubated with antibodies for surface staining for 30 min at 4 °C in the dark. After centrifugation cells were fixed and permeabilized using BD Pharmingen Transcription Factor Buffer set (BD Biosciences, San Jose, CA, USA), followed by intranuclear staining in permeabilization buffer. Antibodies were titrated to determine optimal working dilutions. Final working dilutions and the complete antibody list are provided in Supplementary Table S1. Regulatory T (Treg) cells were defined as CD3^+^CD4^+^FOXP3^+^, Tfh cells as CD3^+^CD4^+^CD45RA^−^CXCR5^+^FOXP3^−^, and Tfr cells as CD3^+^CD4^+^CD45RA^−^CXCR5^+^FOXP3^+^. These operational definitions are commonly applied in peripheral blood [26,28], as circulating Tfh-like cells typically exhibit low or undetectable BCL6 expression compared to their germinal centre counterparts [32].

An overview of the gating strategy is shown in Supplementary Figure S1. Data were acquired using a Navios EX flow cytometer (Beckman Coulter, Brea, CA, USA) and analyzed with FlowJo Software, version 10.9.0 (BD Life Sciences, Ashland, OR, USA). Absolute counts of T cell subpopulations were calculated based on complete blood count (CBC) data obtained on the same day. CBC analysis was performed using electrical impedance and flow cytometry on a Beckman Coulter system.

### 2.6. Statistical Analysis

Normality was assessed using the Shapiro–Wilk test and Q–Q plots. Both indicated deviations from normality; therefore, all group comparisons were performed using non-parametric tests (Mann–Whitney U or Kruskal–Wallis). We used the Mann–Whitney U test for two-group comparisons and the Kruskal–Wallis test for comparisons involving more than two groups. Fisher’s exact test was used to assess association between categorical variables. Correlations between continuous variables were evaluated with the Spearman correlation coefficient. All statistical analyses were performed using GraphPad Prism version 10.10 (Boston, MA, USA), with a significance threshold set at α = 0.05.

## 3. Results

### 3.1. Preserved SARS-CoV-2-Specific Antibody Responses in CLL Patients Despite Reduced Total Immunoglobulin Levels

**Figure 1:**
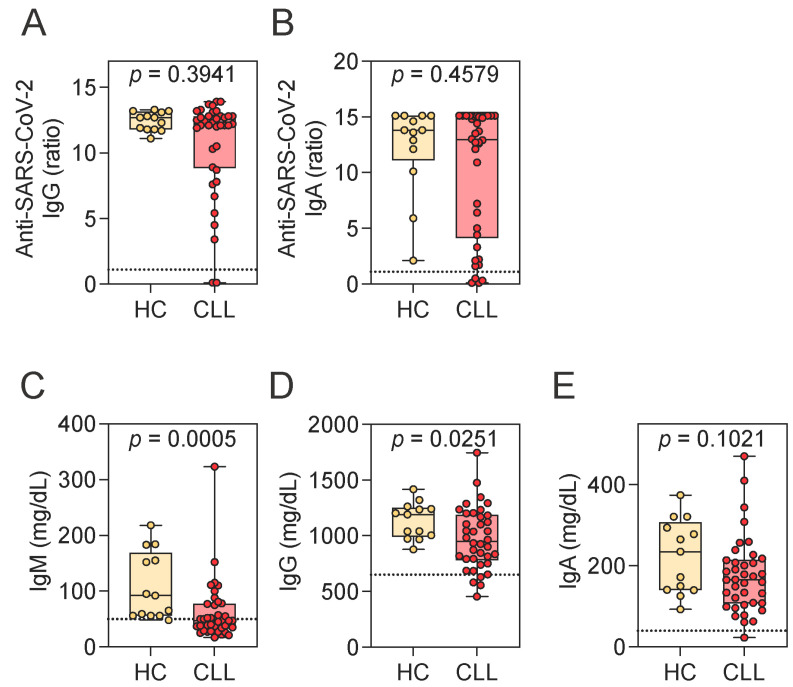
Assessment of SARS-CoV-2-specific and total immunoglobulin levels. (**A**,**B**) SARS-CoV-2-specific IgG (**A**) and IgA (**B**) levels in HCs and CLL patients. (**C**–**E**) Total serum levels of IgM (**C**), IgG (**D**), and IgA (**E**) in HCs and CLL patients. Mann–Whitney U test. Notched box plots represent the 25th and 75th percentile values; horizontal lines represent median values; whiskers indicate minimum and maximum values. In panels (**A**,**B**), the dotted line denotes the positivity for SARS-CoV-2 spike-specific antibodies: ratio > 1.1. The number of CLL patients in each category is as follows: SARS-CoV-2-specific IgG (**A**): 2 negative, 0 borderline, 36 positive; SARS-CoV-2-specific IgA (**B**): 4 negative, 0 borderline, 34 positive. In panels (**C**–**E**), dotted lines indicate lower reference limits for total immunoglobulin levels: 50 mg/dL for IgM, 650 mg/dL for IgG, and 40 mg/dL for IgA. HCs, healthy controls; CLL, chronic lymphocytic leukemia; Ig, immunoglobulin.

Given the frequently observed immune alterations in CLL and the limited understanding of SARS-CoV-2-specific responses in treatment-naive patients, we quantified SARS-CoV-2-specific antibody responses and examined their relationship to levels of total IgM, IgG, and IgA. SARS-CoV-2 spike–specific IgG and IgA levels were comparable between CLL patients and HCs (Figure 1A,B). Indeed, most CLL patients mounted a SARS-CoV-2-specific antibody response, with 94.7% (*n* = 36) positive for IgG and 89.5% (*n* = 34) for IgA.

This robust antibody response was present despite reductions in total immunoglobulin levels, particularly IgM. Both IgM and IgG concentrations were significantly lower in CLL patients compared to HCs, while IgA levels showed a trend toward reduction (Figure 1C–E). Notably, 55.3% (*n* = 21) of patients had total IgM concentrations below the lower limit of the reference range (<50 mg/dL). Reductions below the reference range were less common for IgG (10.5%, *n* = 4; <650 mg/dL) and IgA (2.6%, *n* = 1; <40 mg/dL).

To further investigate whether these reductions in total immunoglobulin levels influenced the magnitude of the virus-specific response, we next examined correlations between total and SARS-CoV-2-specific antibody levels. No significant correlations were observed between SARS-CoV-2-specific IgG levels and total IgG (*r* = 0.285, *p* = 0.083), IgM (*r* = 0.294, *p* = 0.073), or IgA (*r* = 0.058, *p* = 0.729), nor between SARS-CoV-2–specific IgA levels and total IgG (*r* = −0.011, *p* = 0.948), IgM (*r* = 0.062, *p* = 0.712), or IgA (*r* = 0.077, *p* = 0.648). These findings suggest that SARS-CoV-2-specific antibody responses are preserved in CLL patients and appear to be independent of baseline immunoglobulin perturbations. This dissociation was also evident at the individual level, with patient-specific examples illustrating the lack of a consistent relationship between total and SARS-CoV-2-specific immunoglobulin levels. Two CLL patients who were negative for both spike-specific IgG and IgA (Supplementary Table S2) had total IgM levels below the lower reference limit, while their total IgA and IgG levels remained within the normal range. Conversely, two additional CLL patients who failed to mount SARS-CoV-2–specific IgA responses had total IgA, IgM, and IgG levels within reference intervals.

We assessed prior natural exposure to SARS-CoV-2 by measuring IgG antibodies against NCP, which is not induced by mRNA vaccination. The frequency of NCP seropositivity was not significantly different between HCs and CLL patients (Table 2). Of note, no evidence of severe COVID-19 or related complications was documented in either CLL patients or HCs, including those with prior SARS-CoV-2 infection (NCP-seropositive).

Among CLL patients, SARS-CoV-2-specific IgG and IgA levels were significantly higher in NCP-positive individuals compared to those who were NCP-negative (Supplementary Figure S2A,B), suggesting that infection may augment antigen-specific antibody response in CLL. This infection-associated difference was not observed among HCs, in whom prior infection did not further increase antibody levels.

The dissociation between reduced total immunoglobulin levels and preserved virus-specific responses highlights the complexity of humoral immune impairment in early-stage, treatment-naive CLL. While global hypogammaglobulinemia is a hallmark of CLL, our findings suggest that antigen-specific B cell responses can still be mounted, particularly in the context of prior natural infection. This indicates that B cell dysfunction in early CLL may be selective rather than absolute, allowing for partial preservation of vaccine- or infection-induced immunity.

### 3.2. Evidence of Functional T Cell Immunity to SARS-CoV-2 in CLL Patients

**Figure 2:**
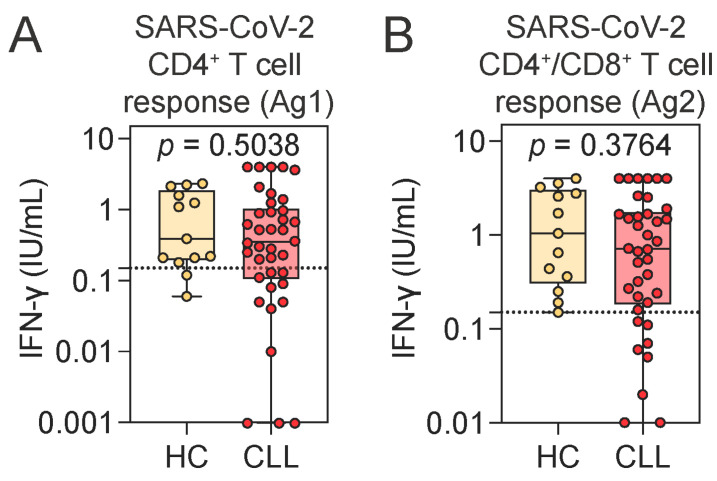
Assessment of SARS-CoV-2-specific T cell responses using an IFN-γ release assay. (**A**,**B**) SARS-CoV-2-specific CD4^+^ T cell (**A**) and combined CD4^+^ and CD8^+^ T cell responses (**B**), measured by ELISA as IFN-γ release following peripheral blood stimulation with SARS-CoV-2-specific peptide antigens in HCs and CLL patients. Mann–Whitney U test. Notched box plots represent the 25th and 75th percentile values; horizontal lines represent median values; whiskers indicate minimum and maximum values. Dotted lines indicate the assay cutoff for SARS-CoV-2-specific T cell response: 0.15 IU/mL. HCs, healthy controls; CLL, chronic lymphocytic leukemia; IFN-γ, interferon gamma.

Given the largely preserved humoral responses, we next investigated whether T cell immunity to SARS-CoV-2 was similarly maintained in CLL. Quantitative assessment of IFN-γ release following stimulation with SARS-CoV-2-derived peptides revealed no significant differences between CLL patients and HCs (Figure 2A,B). A positive T cell response was defined as an IFN-γ level ≥0.15 IU/mL in response to the peptide pool that stimulates both CD4^+^ and CD8^+^ T cells (Ag2 tube). By this criterion, 78.9% (*n* = 30) of CLL patients exhibited a detectable T cell response, while all HCs showed a positive response.

Notably, all CLL patients who lacked a detectable SARS-CoV-2-specific T cell response had seroconverted for SARS-CoV-2 IgG, while two of them had borderline levels of SARS-CoV-2-specific IgA. Conversely, the two CLL patients who were seronegative for both SARS-CoV-2 IgG and IgA demonstrated robust spike-specific T cell responses. Individual-level data are provided in Supplementary Table S2. Importantly, the magnitude of SARS-CoV-2-specific T cell responses did not differ between individuals with or without prior natural exposure to SARS-CoV-2 (as assessed by the presence of anti-NCP IgG). Additionally, the time since the last vaccination was similar across CLL patients with or without a SARS-CoV-2-specific T cell response and HCs (Supplementary Figure S2C–E). No correlation between IFN-γ release and time since vaccination was observed (Supplementary Figure S2F).

These findings underscore the discordance that can occur between antigen-specific antibody and cellular immunity in CLL, yet suggest that functional SARS-CoV-2-specific immunity is still achieved through at least one of these pathways in the majority of cases.

### 3.3. Tfh and Tfr Cells Are Expanded in CLL Patients

To better understand how SARS-CoV-2-specific immunity is preserved in CLL despite reductions in total immunoglobulin levels, we analyzed the CD4^+^ T cell compartment, focusing on Tfh and Tfr cells. These subsets play critical roles in shaping GC responses and antibody production, and are often dysregulated in CLL [26,28].

**Figure 3:**
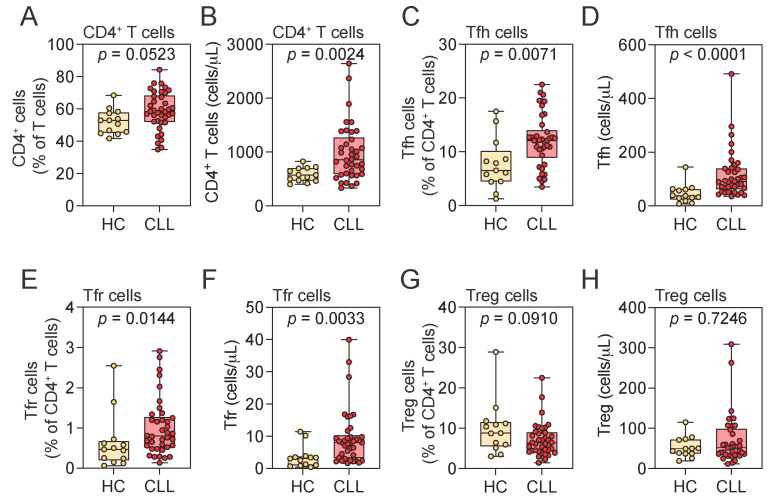
Distribution of CD4^+^ T cell populations in peripheral blood. (**A**–**H**) Frequency and absolute numbers of CD4^+^ T (**A**,**B**), Tfh (**C**,**D**), Tfr (**E**,**F**), and Treg (**G**,**H**) cells in HCs and CLL patients. Mann–Whitney U test. Notched box plots represent the 25th and 75th percentile values; horizontal lines represent median values; whiskers indicate minimum and maximum values. Tfh, T follicular helper; Tfr, T follicular regulatory; Treg, regulatory T cell; HCs, healthy controls; CLL, chronic lymphocytic leukemia.

There was a trend toward increased CD4^+^ T cell frequency in CLL patients compared to HCs (Figure 3A), and the absolute numbers were significantly elevated in the CLL group (Figure 3B). Within this compartment, both the frequency and absolute number of Tfh and Tfr cells were significantly increased in CLL patients relative to HCs (Figure 3C–F). In contrast, the frequency and absolute number of Treg cells were comparable between the groups (Figure 3G,H).

### 3.4. Reduced SARS-CoV-2-Specific T Cell Response Is Associated with Tfh Cell Expansion in CLL

**Figure 4:**
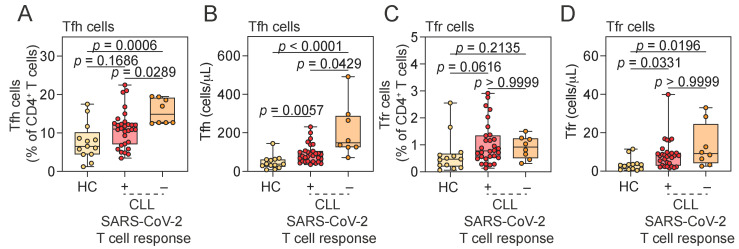
Association between SARS-CoV-2-specific T cell responses and T follicular cell subsets. (**A**–**D**) Frequency and absolute number of Tfh (**A**,**B**) and Tfr (**C**,**D**) cells in HCs and CLL patients stratified by SARS-CoV-2-specific T cell response status. Kruskal–Wallis test. Vertical lines represent the median and 25th and 75th percentile values. Tfh, T follicular helper; Tfr, T follicular regulatory; HCs, healthy controls; CLL, chronic lymphocytic leukemia; IFN-γ, interferon gamma; CLL SARS-CoV-2 T cell response: “+” indicates CLL patients with detectable SARS-CoV-2-specific T cell response (IFN-γ^+^ in response to Ag2); “−” indicates CLL patients lacking such a response.

**Figure 5:**
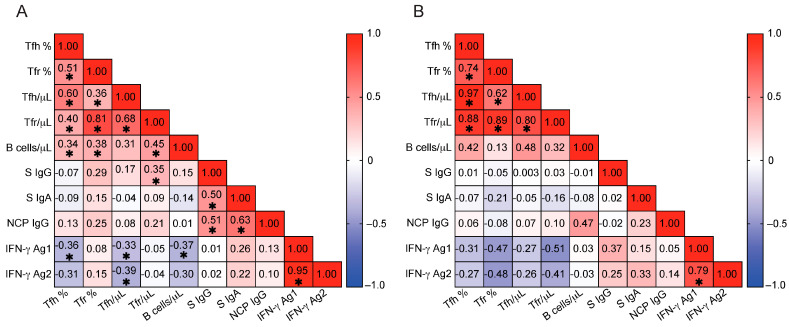
Correlations between T follicular cells, B cells, and SARS-CoV-2-specific immunity. (**A**,**B**) Spearman correlation coefficients are shown for pairwise comparisons between immune parameters in CLL patients (**A**) and HCs (**B**). The strength and direction of correlations are indicated by color intensity and scale (ranging from −1 to +1), with positive correlations in red and negative correlations in blue. Asterisks indicate statistically significant differences (*p* < 0.05). In CLL patients (**A**), Tfh cell parameters show a negative correlation with IFN-γ responses to Ag1 and Ag2 peptide pools. HCs, healthy controls; CLL, chronic lymphocytic leukemia; Tfh %, T follicular helper cell frequency (% of CD4^+^ T cells); Tfr %, T follicular regulatory cell frequency (% of CD4^+^ T cells); Tfh /μL, absolute number of T follicular helper cells (cells/μL); Tfr /μL, absolute number of T follicular regulatory cells (cells/μL); B cells /μL, absolute number of CD19^+^ B cells (cells/μL); S IgG, serum IgG antibodies to SARS-CoV-2 spike protein (ratio); S IgA, serum IgA antibodies to SARS-CoV-2 spike protein (ratio); NCP IgG, serum IgG antibodies to SARS-CoV-2 nucleocapsid protein (ratio); IFN-γ, interferon gamma; IFN-γ Ag1, IFN-γ release in response to SARS-CoV-2 Ag1 peptide pool (IU/mL); IFN-γ Ag2, IFN-γ release in response to SARS-CoV-2 Ag2 peptide pool (IU/mL).

We next evaluated the relationship between elevated Tfh and Tfr cell frequencies in CLL patients and their SARS-CoV-2-specific immune responses. We compared HCs (all with detectable SARS-CoV-2-specific T cell response) with CLL patients stratified by the presence or absence of virus-specific T cell immunity, as measured by IFN-γ release assay. Patients were stratified specifically by Ag2 responses, as this peptide pool stimulates both CD4^+^ and CD8^+^ T cells and provides a broader measure of virus-specific cellular immunity. Tfh cell frequencies showed a trend toward higher levels in CLL patients who mounted a SARS-CoV-2-specific T cell response compared to HCs (*p* = 0.1686), and absolute Tfh cell numbers were increased (Figure 4A). Paradoxically, both the frequency and absolute number of Tfh cells were significantly elevated in CLL patients who failed to mount a virus-specific T cell response, compared to both responders and HCs (Figure 4A,B). Indeed, both Tfh frequency and absolute number negatively correlated with antigen-specific T cell responses in CLL (Figure 5). In contrast, Tfr cell frequencies and absolute numbers showed no consistent relationship with SARS-CoV-2-specific T cell response status in CLL patients (Figure 4C,D). Whereas Tfh frequency and number clearly distinguished CLL responders from non-responders, no significant differences were detected in total B cell counts, total lymphocytes, or baseline serum IgM and IgG levels between the two groups (Supplementary Figure S3A–D). This indicates that impaired virus-specific T cell immunity in non-responders is not attributable to general differences in lymphocyte composition or serum immunoglobulin levels, but rather may be associated with a more specific dysregulation of the Tfh cell compartment.

To account for cohort heterogeneity in vaccination history, we performed a sensitivity analysis excluding the six CLL patients who had received only a single vaccine dose. The main findings were unchanged: Tfh cell frequency remained significantly higher in non-responders compared to responders and HCs, and absolute Tfh numbers showed a similar trend (*p* = 0.065 vs. *p* = 0.0429 in the full cohort; Supplementary Figure S4A–D). Notably, only one of the six single-dose patients was a non-responder, while the remaining five were responders, indicating that the key findings were not driven by this subgroup. Thus, restricting the cohort to individuals who received ≥2 doses confirmed that the relationship between impaired SARS-CoV-2-specific T cell responses and elevated Tfh cell frequencies is robust and not dependent on the six single-dose participants.

The absolute number of Tfr cells showed a correlation with SARS-CoV-2-specific IgG levels in CLL patients but not in HCs. However, neither the frequency nor absolute number of Tfh cells was associated with antigen-specific immunoglobulin responses in either CLL patients or HCs (Figure 5).

To explore why certain CLL patients failed to mount SARS-CoV-2-specific T cell responses, we examined the relationship between Tfh cell expansion and total B cell abundance. Total B cell counts were used to allow direct comparisons with HCs. In untreated CLL, the leukemic clone accounts for the vast majority of circulating CD19^+^ B cells, whereas in HCs, CD19^+^ cells represent a polyclonal population of non-malignant B cells. Thus, total CD19^+^ counts in CLL primarily reflect malignant B cell burden and provide a relevant parameter to investigate T cell-B cell interactions in this disease. In CLL patients, the frequencies of both Tfh and Tfr cells positively correlated with peripheral CD19^+^ B cell numbers (Tfh %, r = 0.336, *p* = 0.039; Tfr %, r = 0.379, *p* = 0.019; Tfh absolute, r = 0.307, *p* = 0.061 [trend]; Tfr absolute, r = 0.449, *p* = 0.005), while no such associations were observed in HCs. These findings suggest that in CLL, expansion of Tfh and Tfr cells may be shaped, at least in part, by the malignant B cell compartment. Together, these data support a model in which Tfh cells in CLL are more likely expanded in response to or in support of malignant B cells, rather than to coordinate productive antiviral immunity. This skewing may limit their availability, thereby decoupling Tfh expansion from the coordination of effective antiviral effector responses, as observed in our cohort. Prognostic factors such as elevated β2M or advanced Binet stage were not associated with impaired SARS-CoV-2-specific T cell responses (Ag2) (Supplementary Figure S4E,F), suggesting that these classical factors do not account for the antigen-specific T cell dysregulation observed in our cohort.

## 4. Discussion

This study provides new insights into the immune competence of treatment-naive CLL patients by demonstrating that SARS-CoV-2-specific antibody and T cell responses can be preserved despite hallmark features of immune dysregulation. Although CLL is characterized by hypogammaglobulinemia and T cell subset changes, most patients still mounted robust antigen-specific IgG and IgA responses following SARS-CoV-2 vaccination, with titers comparable to those of HCs. Virus-specific T cell responses were also detected in most patients. These findings suggest that, despite systemic immune alterations in CLL, adaptive responses to novel antigens can still be generated. However, the decoupling between Tfh expansion and virus-specific T cell immunity—most pronounced in patients lacking detectable anti-SARS-CoV-2-specific T cell responses—is consistent with the hypothesis that disease-intrinsic factors may skew T cell-B cell interactions toward supporting malignant B cells rather than promoting effective antiviral coordination. Alternative explanations, such as functional exhaustion or anergy of the expanded Tfh populations, may also contribute to this dissociation and warrant further investigation.

CLL patients are at high risk for severe COVID-19 outcomes [9,10]. Early reports during the pandemic indeed indicated that CLL patients had impaired antibody responses to SARS-CoV-2 infection or vaccination [11,12,13,14,15]. However, our findings suggest that many untreated CLL patients retain a substantial capacity to mount SARS-CoV-2-specific immunity. We observed high rates of seroconversion and robust T cell responses in most treatment-naive patients, indicating a more preserved adaptive immune function than previously appreciated. Notably, this preserved responsiveness occurred despite significantly reduced total immunoglobulin levels (IgG and IgM) and expanded follicular cell subsets, reflecting baseline hypogammaglobulinemia and T cell compositional alterations common in CLL. These results underscore a disconnect between global immune composition and antigen-specific function in CLL, highlighting that low immunoglobulin levels or altered immune cell subsets do not necessarily preclude effective vaccine- or infection-induced immunity. This has important implications for how immune competence is evaluated in patients with CLL.

A critical factor influencing immune response in CLL is treatment status. In previous studies, failure to mount SARS-CoV-2-specific immune responses was associated with immunosuppressive treatment, such as Bruton tyrosine kinase inhibitors or anti-CD20 monoclonal antibodies [9,10,13,33]. In contrast, repeated antigen exposure may help compensate for immune dysregulation. Emerging studies suggest that repeated exposure to SARS-CoV-2 antigens—particularly through booster vaccination—can improve seroconversion rates in this population [15,16,34,35]. In our cohort, approximately half of both CLL patients and HCs had detectable anti-NCP IgG, indicating natural exposure. Notably, CLL patients with prior COVID-19 infection mounted stronger spike-specific antibody responses (Supplementary Figure S2A,B), further supporting the idea that cumulative antigenic exposure enhances immunity. The relatively long median interval since last vaccination in CLL patients (312.5 days) also suggests durability—alongside magnitude—of the humoral response, consistent with recent data on antibody persistence in this population [12,36]. Thus, discrepancies between our findings and prior studies may reflect differences in treatment status or cumulative antigenic exposure from prior infection and repeated vaccination.

T cell immunity is a key correlate of protection from severe COVID-19 and displays greater resilience across viral variants than circulating antibodies [36,37,38]. Lack of T cell responsiveness may be clinically important: impaired CD4^+^ T cell responses are linked to higher COVID-19 mortality [37], whereas preserved CD8^+^ T cells correlate with better outcomes even when antibodies are reduced [33]. In hematologic malignancies, including CLL, robust virus-specific T cell responses can partly compensate when antibody responses are weak, whereas diminished T cell responses are associated with worse clinical outcomes [33]. In our study a SARS-CoV-2-specific T cell response was detected in 78.9% of treatment-naive patients, similar in magnitude to HCs. However, patients without a T cell response had seroconverted for IgG. This finding is striking, as B cell dysfunction is generally more profound than T cell impairment in CLL. Importantly, classical prognostic markers such as β2 microglobulin (β2M) levels and Binet stage were not associated with impaired SARS-CoV-2-specific T cell responses in our cohort, suggesting that antigen-specific T cell dysregulation arises independently of established disease stage indicators.

An effective antibody response typically relies on coordinated T cell help, particularly from Tfh cells. In immunocompetent individuals, the induction of Tfh cells is one of the initial adaptive immune responses to viral antigens such as SARS-CoV-2, and plays a central role in promoting neutralizing antibody production [38]. In CLL patients, however, Tfh cells are often expanded and dysfunctional [23,24,25,26,27,28]. These Tfh cells have been previously suggested to support malignant B cells. We also observed significantly increased Tfh frequencies and absolute numbers in our CLL cohort. Supporting Tfh interactions with malignant B cells, we observed correlations with B cells and both Tfr and Tfh cells in CLL patients but not in HCs. Notably, patients who failed to mount a SARS-CoV-2-specific T cell response exhibited the highest levels of Tfh cells. This finding supports the hypothesis that expanded Tfh populations may reflect tumor-driven immune skewing rather than effective antiviral coordination. However, it is also possible that these Tfh cells are functionally exhausted, anergic, or otherwise impaired in their ability to support antiviral responses. The mechanistic basis for this dissociation remains unclear but may involve complex tumor-driven immune dysregulation affecting T cell priming, differentiation, or effector function.

Our data suggest a decoupling of Tfh activation from effective antiviral T cell immunity and motivate deeper phenotyping of Tfh subsets and lymph-node microenvironments in CLL. While this study focused on the overall frequencies and absolute numbers of circulating Tfh and Tfr cells, a phenotypic analysis of Tfh subsets (e.g., Tfh1, Tfh2, Tfh17), functional markers (e.g., ICOS, PD-1, CD25), and their transcriptional profiles were not performed. These parameters may provide additional mechanistic insights into the polarization and functional state of Tfh cells in CLL. We acknowledge this as a limitation and suggest that future studies incorporating subset-specific and functional markers, as well as transcription factor profiling could further elucidate the role of Tfh heterogeneity in shaping antiviral immunity versus tumor interactions.

## 5. Conclusions

Treatment-naive CLL patients retain substantial capacity to mount SARS-CoV-2-specific antibody and T cell responses after vaccination, despite reduced immunoglobulin levels and altered T cell subsets. In CLL patients, failure to mount a SARS-CoV-2-specific T cell response is coupled with substantial Tfh expansion. This expansion also correlates with malignant B cell abundance, potentially reflecting skewing by the leukemic clone or chronic antigen-driven exhaustion. Together, these findings reveal a previously underappreciated interplay between T cell compartments in CLL, whereby pronounced Tfh expansion associates with impaired virus-specific T cell responses.

## Figures and Tables

**Table 1:** Demographic, clinical, and laboratory characteristics of study participants.

Variables ^1^	Healthy Controls (*n* = 13)	CLL Patients (*n* = 38)	*p* Value ^2^
Sex (*n*, %)			>0.9999
Male	8 (61.5%)	24 (63.2%)	
Female	5 (38.5%)	14 (36.8%)	
Age (years)	66 (64–69.5)	70 (61.8–76.3)	0.3251
Binet stage (*n*, %)			
A	-	29 (76.3%)	
B	-	9 (23.7%)	
WBC (×10^9^/L)	5.70 (5.10–6.40)	17.55 (13.68–22.25)	<0.0001
Lymphocytes (×10^9^/L)	1.90 (1.45–2.05)	11.40 (8.50–17.78)	<0.0001
B cells (×10^9^/L)	0.10 (0.06–0.13)	7.04 (4.42–13.24)	<0.0001
Platelets (×10^9^/L)	235.0 (201.5–258.5)	207.0 (160.8–243.8)	0.0601
HGB (g/L)	140 (132–151)	144 (132–152)	0.6189
Total protein (g/L)	69.3 (66.9–71.5)	70.7 (67.8–73.1)	0.3111
Genetic alterations (*n*, %)			
Del 11q22.3	-	6/31 (19.4%)	
Del 17q13.1	-	1/31 (3.2%)	
Trisomy 12	-	2/27 (7.4%)	
Del 13q14.2-q14.3	-	21/31 (67.7%)	
Number of SARS-CoV-2 vaccine doses received (*n*, %)			0.4739
4 doses	2 (15.4%)	5 (13.2%)	
3 doses	8 (61.5%)	16 (42.1%)	
2 doses	3 (23.1%)	11 (28.9%)	
1 dose	0	6 (15.8%)	
Time since last vaccination (days)	153 (114.0–480.5)	312.5 (179.0–480.3)	0.2492

^1^ Data are median and interquartile range. ^2^ Mann–Whitney U test was used for continuous variables and Fisher’s exact test for categorical variables. CLL, chronic lymphocytic leukemia; WBC, white blood cell; HGB, hemoglobin.

**Table 2:** Levels of anti-SARS-CoV-2 NCP IgG antibodies in CLL patients and healthy controls.

	Healthy Controls (*n* = 13)	CLL Patients (*n* = 38)	*p* Value *
Anti-SARS-CoV-2 NCP IgG status (*n*, %)			0.6903
Negative	5 (38.5%)	19 (50.0%)	
Borderline	1 (7.7%)	2 (5.3%)	
Positive	7 (53.8%)	17 (44.7%)	

* Fisher’s exact test. CLL, chronic lymphocytic leukemia; NCP, nucleocapsid protein.

## Data Availability

The original contributions presented in this study are included in the article/Supplementary Materials. Further inquiries can be directed to the corresponding author.

## Supplementary Materials

The following supporting information can be downloaded at: https://www.mdpi.com/article/10.3390/pathogens14090890/s1, Figure S1: Gating strategy for T lymphocyte analysis; Figure S2: SARS-CoV-2-specific antibody and T cell responses in HCs and CLL patients; Figure S3: Association between SARS-CoV-2-specific T cell responses and total B cell counts, lymphocyte counts, and serum levels of immunoglobulin G and M; Figure S4: SARS-CoV-2-specific T cell responses in HCs and CLL patients with ≥2 vaccine doses. Table S1: Antibody panel for T cell phenotyping; Table S2: Individual-level data on SARS-CoV-2-specific immune responses in CLL patients and HCs.

## Abbreviations

The following abbreviations are used in this manuscript:

β2M	Beta 2 microglobulin
BCL6	B cell lymphoma 6
CBC	Complete blood count
CD	Cluster of differentiation
CLL	Chronic lymphocytic leukemia
COVID-19	Coronavirus disease 2019
CXCR5	C-X-C chemokine receptor type 5
ELISA	Enzyme-linked immunosorbent assay
FcR	Fc receptor
FOXP3	Forkhead box P3
GC	Germinal center
HCs	Healthy controls
HGB	Hemoglobin
Ig	Immunoglobulin
IGRA	Interferon gamma release assay
IFN-γ	Interferon gamma
NCP	Nucleocapsid protein
PBMC	Peripheral blood mononuclear cells
SARS-CoV-2	Severe acute respiratory syndrome coronavirus 2
Tfh	T follicular helper
Tfr	T follicular regulatory
Treg	Regulatory T cell
WBCs	White blood cells
