# Supplementary material for: Attenuated SARS-CoV-2-Specific T Cell Responses Are Associated with T Follicular Helper Cell Expansion in Treatment-Naive Chronic Lymphocytic Leukemia Patients
Source: Pathogens. 2025 Sep 5;14(9):890. doi: 10.3390/pathogens14090890 (PMC12472264; doi:10.3390/pathogens14090890)
Supplement: Supplementary file 1 [file pathogens-14-00890-s001.zip › pathogens-3812028-supplementary.pdf]

Supplementary Materials
Attenuated SARS-CoV-2-specific T cell responses are associated with T follicular helper cell expansion in treatment-naive chronic lymphocytic leukemia patients
Baiba Šlisere *et al.*

**Antigen**	**Fluorochrome**	**Clone**	**Cat.Nr.**	**Supplier**	**Dilution**
CXCR5	FITC	J252D4	356914	BioLegend	1:20
CD3	PECy5	UCHT1	300410	BioLegend	1:50
CD4	APC	OKT4	317416	BioLegend	1:20
CD45RA	AF700	HI100	56-0458-42	Invitrogen	1:20
FOXP3	PE	206D	320108	BioLegend	1:20

Supplementary Table S1. Antibody panel for T cell phenotyping.

**ID**	**Spike IgG (ratio)**	**Spike IgG status**	**Spike IgA (ratio)**	**Spike IgA status**	**Total IgM (mg/dL)**	**Total IgG (mg/dL)**	**Total IgA (mg/dL)**	**NCP IgG (ratio)**	**NCP IgG status**	**IFN-γ Ag1 (IU/mL)**	**IFN-γ Ag2 (IU/mL)**
CLL-001	0,1	Negative	0,1	Negative	22	629	180	0,1	Negative	4	4
CLL-002	7,6	Positive	14,8	Positive	26	739	218	0,1	Negative	4	4
CLL-003	8,9	Positive	2,2	Positive	78	793	132	0,1	Negative	0	0,16
CLL-004	5,4	Positive	4,4	Positive	100	873	239	0	Negative	0,89	1,5
CLL-005	12,8	Positive	12,1	Positive	25	1100	344	0,2	Negative	0,3	0,7
CLL-006	12,4	Positive	12,6	Positive	39	1123	96	0,1	Negative	0,01	0,02
CLL-007	12,7	Positive	3,3	Positive	152	1236	191	0,1	Negative	0,25	0,38
CLL-008	12,3	Positive	6,4	Positive	37	817	23	6,9	Positive	1,4	2,53
CLL-009	10,3	Positive	15,1	Positive	27	790	108	0,1	Negative	0,34	0,51
CLL-010	12,1	Positive	14,4	Positive	88	1293	165	6,6	Positive	4	4
CLL-011	13,1	Positive	15,1	Positive	47	1233	257	5,6	Positive	4	4
CLL-012	13,9	Positive	15,1	Positive	110	1104	410	5,1	Positive	0,97	1,67
CLL-013	12,2	Positive	15	Positive	17	684	78	0,9	Borderline	0,28	1,68
CLL-014	12,1	Positive	12,9	Positive	45	652	90	1,5	Positive	0,11	0,12
CLL-015	11,9	Positive	12,7	Positive	29	1288	166	0,1	Negative	1,7	1,48
CLL-016	12,1	Positive	10,9	Positive	35	934	160	0,2	Negative	3,63	4
CLL-017	12,5	Positive	15,1	Positive	115	1201	186	1,6	Positive	2,08	4
CLL-018	6,7	Positive	15,1	Positive	50	834	121	5,2	Positive	0,36	1
CLL-019	13,7	Positive	13	Positive	37	1475	109	1,8	Positive	0,2	0,27
CLL-020	0,1	Negative	0,1	Negative	21	814	104	0	Negative	0,62	2,62
CLL-021	10,5	Positive	1,7	Positive	47	964	227	0,1	Negative	0,13	0,19
CLL-022	12	Positive	15,1	Positive	27	901	308	5,4	Positive	0,72	0,72
CLL-023	12,8	Positive	15,1	Positive	40	454	99	3,8	Positive	0,05	0,06
CLL-024	12,6	Positive	15,1	Positive	38	983	214	6,8	Positive	0,05	0,11
CLL-025	13,3	Positive	15,1	Positive	323	846	174	0,2	Negative	0,93	1,91
CLL-026	13,6	Positive	13,7	Positive	75	1346	259	7,1	Positive	0,21	0,24
CLL-027	12,1	Positive	13,5	Positive	55	1169	179	8,3	Positive	1,25	1,57
CLL-028	3,4	Positive	5	Positive	81	1183	207	0,7	Negative	0,08	0,07
CLL-029	8,7	Positive	1,6	Positive	28	685	76	0,1	Negative	0,04	0,05
CLL-030	4,5	Positive	0,3	Negative	52	1204	201	0,1	Negative	0	0
CLL-031	7,8	Positive	0,5	Negative	57	1041	208	0,2	Negative	0	0
CLL-032	13,2	Positive	15	Positive	111	1044	158	0,8	Borderline	0,13	0,22

CLL-033	12,1	Positive	15,1	Positive	34	750	149	1,8	Positive	0,67	0,86
CLL-034	13,2	Positive	15,1	Positive	38	556	63	2,5	Positive	0,53	1,26
CLL-035	12,8	Positive	7,2	Positive	47	580	61	0,1	Negative	0,52	1,39
CLL-036	12,8	Positive	12,7	Positive	50	1038	134	1,5	Positive	0,09	0,32
CLL-037	13,9	Positive	14,9	Positive	77	1746	470	7,2	Positive	0,23	0,55
CLL-038	12,7	Positive	2,1	Positive	52	925	134	0,1	Negative	0,52	0,67
HC-001	13,1	Positive	15,1	Positive	59	1039	93	0,1	Negative	1,59	4
HC-002	13,2	Positive	14,6	Positive	184	1202	141	3,9	Positive	0,39	0,65
HC-003	12,8	Positive	5,9	Positive	218	877	150	0,1	Negative	2,33	3,24
HC-004	11,7	Positive	12,1	Positive	65	1190	172	4,2	Positive	1,1	1,04
HC-005	11,8	Positive	15,1	Positive	155	1236	294	4,5	Positive	2,14	2,78
HC-006	12,7	Positive	10,1	Positive	152	1417	235	1,7	Positive	0,06	0,15
HC-007	12,7	Positive	15,1	Positive	56	1263	374	0,7	Negative	1,25	1,71
HC-008	11,9	Positive	15,1	Positive	92	973	126	0,8	Borderline	0,18	0,25
HC-009	13,2	Positive	12,9	Positive	183	1321	278	1,9	Positive	0,21	0,44
HC-010	11,1	Positive	13,8	Positive	56	1004	321	3,3	Positive	0,12	2,58
HC-011	11,8	Positive	13,6	Positive	48	1240	265	0,6	Negative	0,21	0,19
HC-012	12,3	Positive	2,1	Positive	95	968	140	0,1	Negative	0,22	0,36
HC-013	13,3	Positive	13,8	Positive	56	1041	321	10,5	Positive	2,25	3,57

Supplementary Table S2. Individual-level data on SARS-CoV-2-specific immune responses in CLL patients and HCs. Interpretation of the spike-specific IgG and IgA ratio: negative < 0.8; borderline 0.8 – 1.1; positive >1.1. Reference limits for total immunoglobulin levels: 50 – 300 mg/dL for IgM, 650 – 1600 mg/dL for IgG, and 40 – 350 mg/dL for IgA. HC, healthy controls; CLL, chronic lymphocytic leukemia; Ig, immunoglobulin; NCP, nucleocapsid protein; IFN-γ, interferon γ; IFN-γ Ag1, interferon-γ release in response to SARS-CoV-2 Ag1 peptide pool; IFN-γ Ag2, interferon-γ release in response to SARS-CoV-2 Ag2 peptide pool.

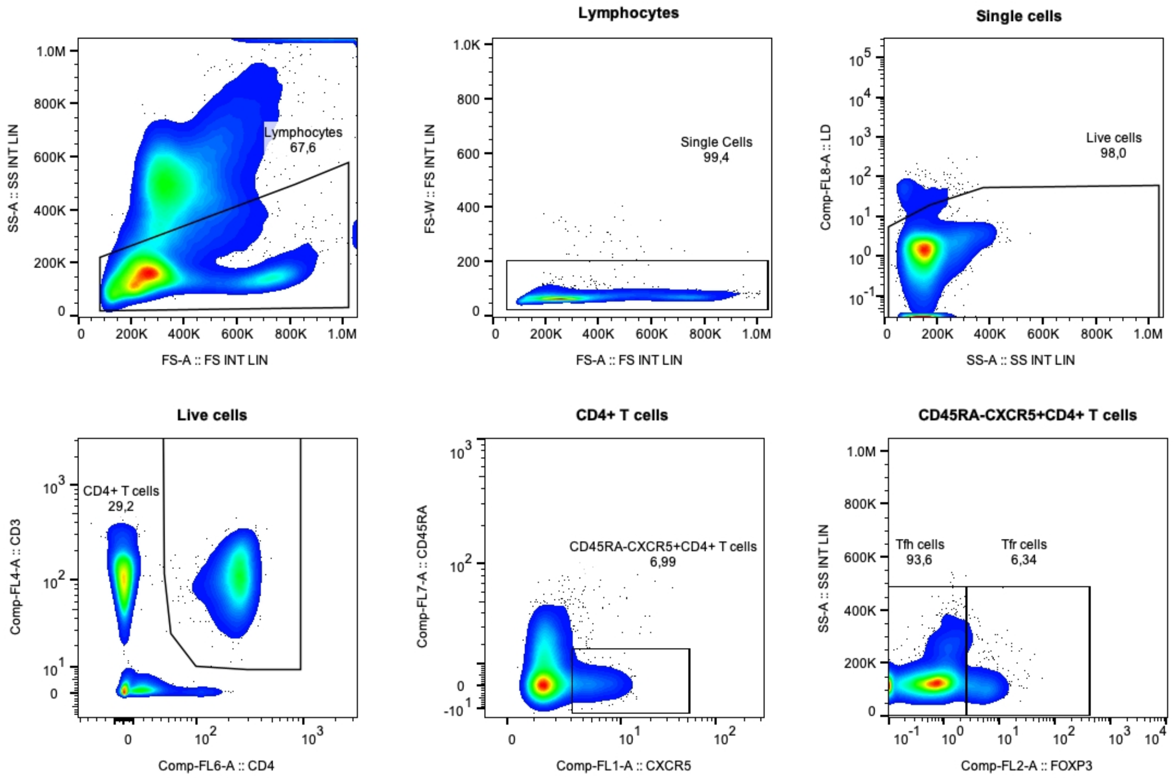

Supplementary Figure S1. Gating strategy for T lymphocyte analysis. Lymphocytes were identified by forward and side scatter (FS-A vs SS-A). Single cells were selected on FS-W vs FS-A to exclude doublets. Viable cells were gated by exclusion of dead cells using a fixable viability dye. From viable lymphocytes, $CD3^{+}CD4^{+}$ T cells were selected. Conventional regulatory T cells (Tregs) were defined as $CD3^{+}CD4^{+}FOXP3^{+}$.

Within CD3⁺CD4⁺ T cells, CD45RA⁻CXCR5⁺ cells were identified as T follicular cells. Among these, T follicular helper (Tfh) cells were defined as CD3⁺CD4⁺CD45RA⁻CXCR5⁺FOXP3⁻, and T follicular regulatory (Tfr) cells as CD3⁺CD4⁺CD45RA⁻CXCR5⁺FOXP3⁺. Representative plots illustrate the gating sequence used for all analyses.

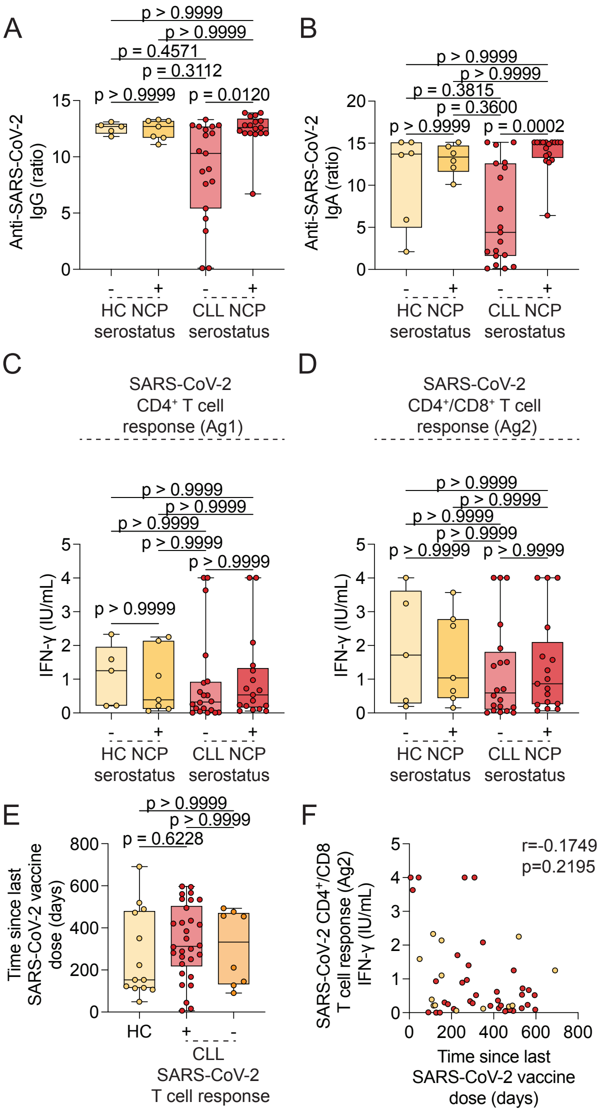

Supplementary Figure S2. SARS-CoV-2-specific antibody and T cell responses in HCs and CLL patients. (**a**, **b**) Levels of SARS-CoV-2-specific IgG (**a**), IgA (**b**) in HCs (n = 12) and CLL patients (n = 36). Groups are stratified by anti-NCP IgG serostatus: "+" indicates individuals with detectable anti-NCP IgG (evidence of prior SARS-CoV-2 infection), and "-" indicates individuals lacking anti-NCP IgG. Borderline anti-NCP cases were excluded. (**c**, **d**) SARS-CoV-2-specific T cell responses to Ag1 (**c**) and Ag2 (**d**) in HCs and CLL patients, grouped by anti-NCP IgG serostatus. (**e**) Time since last SARS-CoV-2 vaccine dose (in days) in HCs and CLL patients, stratified by T cell response status. T cell response was assessed by IFN-γ production in response to Ag2 stimulation: "+" indicates detectable SARS-CoV-2-specific T cell response; "−" indicates no response. Kruskal-Wallis test. (**f**) Correlation between time since last SARS-CoV-2 vaccination (days) and SARS-CoV-2–specific T cell responses (Ag2 IFN-γ release, IU/mL) in HCs and CLL patients. Notched box plots represent the 25th and 75th percentile values; horizontal lines represent median values; whiskers indicate minimum and maximum values. HC, healthy controls; CLL, chronic lymphocytic leukemia; NCP, nucleocapsid protein.

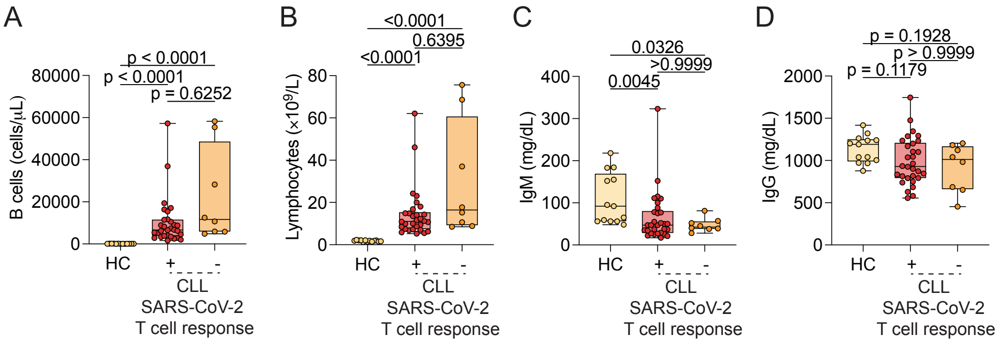

Supplementary Figure S3. Association between SARS-CoV-2-specific T cell responses and total B cell counts, lymphocyte counts, and serum levels of immunoglobulin G and M. (**a**) Absolute number of B cells, (**b**) lymphocytes, and total (**c**) IgM and (**d**) IgG levels in HCs (n = 12) and CLL patients (n = 36). Groups are stratified by SARS-CoV-2-specific T cell response status: "+" indicates CLL patients with detectable SARS-CoV-2-specific T cell response (interferon γ^{+} in response to Ag2); "−" indicates CLL patients lacking such a response. Kruskal-Wallis test. Notched box plots represent the 25th and 75th percentile values; horizontal lines represent median values; whiskers indicate minimum and maximum values. HC, healthy controls; CLL, chronic lymphocytic leukemia; Ig, immunoglobulin.

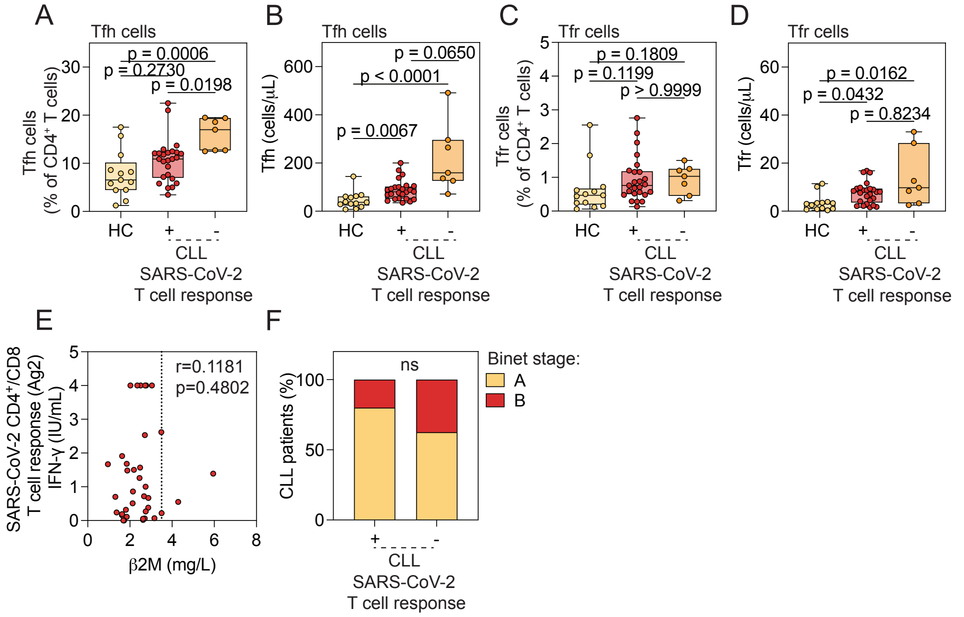

Supplementary Figure S4. SARS-CoV-2-specific T cell responses in HCs and CLL patients with ≥2 vaccine doses. (**a-d**) Frequency and absolute number of Tfh (**a, b**) and Tfr (**c, d**) cells in HCs and CLL that received **≥2 vaccine doses** stratified by SARS-CoV-2-specific T cell response status. (**e**) Correlation between β2-microglobulin (β2M, mg/L) and SARS-CoV-2–specific T cell responses (Ag2 IFN-γ release, IU/mL) in CLL patients. (**f**) Distribution of Binet stage in CLL patients stratified by SARS-CoV-2-specific T cell response status (Ag2). Bars represent the percentage of patients in each stage within responder and non-responder groups. Kruskal-Wallis test. Vertical lines represent the median and 25th and 75th percentile values. Tfh, T follicular helper; Tfr, T follicular regulatory; HC, healthy controls; CLL, chronic lymphocytic leukemia; IFN-γ, interferon γ; CLL SARS-CoV-2 T response: "+" indicates CLL patients with detectable SARS-CoV-2-specific T cell response (IFN-γ^{+} in response to Ag2); "−" indicates CLL patients lacking such a response.
